# Does Reviewing Lead to Better Learning and Decision Making? Answers from a Randomized Stock Market Experiment

**DOI:** 10.1371/journal.pone.0037719

**Published:** 2012-05-30

**Authors:** Patrick Wessa, Ian E. Holliday

**Affiliations:** 1 Leuven Institute for Research on Information Systems, K.U. Leuven, Leuven, Belgium; 2 Aston Brain Centre, Aston University, Aston Triangle, Birmingham, United Kingdom; University of Minnesota, United States of America

## Abstract

**Background:**

The literature is not univocal about the effects of Peer Review (PR) within the context of constructivist learning. Due to the predominant focus on using PR as an assessment tool, rather than a constructivist learning activity, and because most studies implicitly assume that the benefits of PR are limited to the reviewee, little is known about the effects upon students who are required to review their peers. Much of the theoretical debate in the literature is focused on explaining *how* and *why* constructivist learning is beneficial. At the same time these discussions are marked by an underlying presupposition of a causal relationship between reviewing and deep learning.

**Objectives:**

The purpose of the study is to investigate whether the writing of PR feedback causes students to benefit in terms of: perceived utility about statistics, actual use of statistics, better understanding of statistical concepts and associated methods, changed attitudes towards market risks, and outcomes of decisions that were made.

**Methods:**

We conducted a randomized experiment, assigning students randomly to receive PR or non–PR treatments and used two cohorts with a different time span. The paper discusses the experimental design and all the software components that we used to support the learning process: Reproducible Computing technology which allows students to reproduce or re–use statistical results from peers, Collaborative PR, and an AI–enhanced Stock Market Engine.

**Results:**

The results establish that the writing of PR feedback messages causes students to experience benefits in terms of Behavior, Non–Rote Learning, and Attitudes, provided the sequence of PR activities are maintained for a period that is sufficiently long.

## Introduction

### Background

Due to the rapid advance in computer technology, Peer Review (PR) has become an important practice in higher education in a wide variety of fields and educational settings [1]. There are several types of PR but, in general, it can be used as a learning or an assessment tool: *“As a learning tool, assessing their peers can provide students with skills to form judgements about what constitutes high-quality work (…). As an assessment tool, peer assessment can provide teachers with a more accurate picture of individual performance in group work (…).”* [2].

Some educators and educational researchers perceive PR as a formative assessment and grading tool rather than a collaborative learning activity [3] rooted in the traditions of pedagogical constructivism, experiential learning, learner autonomy and similar concepts [4]. The review study of Tillema, Leenknecht and Segers [5] explains how the *“…changing perspective on assessment purposes (i.e., from assessment of learning to assessment for learning)…”* plays an important role for the learner who receives the feedback (i.e. the reviewee): *“Assessment for learning (…), interpreted as providing (in)formative feedback (…), is regarded as a key route in accomplishing significant improvements in students ability in learning how to learn. To promote such learning, assessments prime function is to endorse adaptive, student focused feedback on the learning progress of the learner (…)”* [5]. In other words, *“…peer assessment, …, is a tool especially suited to increase student involvement in classroom assessment”* with *“positive effects on motivation and engagement in learning of students”* [5].

However, and even if it is primarily viewed as a formative incentive, PR practices may restrict a learner's freedom to experiment, to be creative and to collaborate in the joint construction of knowledge and the negotiation of alternatives through debate and argumentation [3]. Indeed, if PR marks are deemed to be important and if they count towards the final grade, then students may engage in copying and other free–riding behaviour, rather than taking the time to develop their non–rote learning skills. On top of that, and even when peer assessments are used on a regular (weekly) basis, it is not necessarily possible for students to detect the free–riding behavior of their peers as is demonstrated in a recent fraud detection study [6] which proposes a technological solution to support reproducibility of research results that are produced by students and to allow us to detect certain types of fraud in assignment–based learning.

In contrast to PR as a formative grading tool, little is known about the effects upon students who are required to review their peers because almost all empirical PR studies focus on the effect on the receiver of the feedback, i.e. the reviewee [7]. One notable exception is a study that investigates the benefits to both the receiver and the reviewer [8]. The empirical findings in this particular study show that the reviewer benefits more than the receiver. Though perhaps surprising at first sight, this observation makes sense if the process of writing peer reviews involves higher order cognitive skills that encourage deep learning. In contrast, receiving review messages may or may not involve actions that have an impact on learning or thinking. The e-learning tools we use, however, cannot measure what happens with feedback messages that are received, e.g., opening a web page does not necessarily imply intensive reading and comprehension.

Most importantly, and *“Despite peer assessment's popularity and advantages, one major problem remains unresolved. At present it is impossible to make claims about what exactly constitutes effective peer assessment; in other words, which peer assessment measures benefit student learning and yield satisfactory psychometric qualities such as reliability and validity.”* [2]. Furthermore, *“… it does complicate the drawing of inferences about causes and effects. This is because the literature usually describes peer assessment in a holistic fashion, that is, without specifying all the variables present in terms of conditions, methods and outcomes.”* [2].

Nevertheless, in the literature, there seems to be a theory-driven belief that PR activities stimulate constructivist learning — or in other words [9], that peer assessment is *“an interactive and communicative process in the service of learning”* and *“a cyclical and interactive process”*. On the other hand, the benefits of PR to either the reviewer or the reviewee are not generally accepted and still engender a lot of debate [4]: *“Literature reviews […] indicate that although various studies seem to have found positive effects of peer assessment on learning, the results are still inconclusive. Moreover, it is unclear under what conditions peer assessment is effective.”*

Even though the process of PR may seem to play an important role, as a formative assessment tool or as a constructivist learning activity, we cannot neglect the fact that there are only few studies in which the effects on learning outcomes are actually tested [7], [9], [4], [2]. In addition, in our literature search, we found no hard empirical evidence to support the hypothesis that writing PR feedback (rather than receiving it) has any beneficial and causal impact on learning. It is this “causality assumption” which lies at the heart of the problem if one wishes to study the impact of PR on learning by means of traditional methodology such as correlational analysis or regression models.

Fortunately, the availability of various e–learning tools that we developed [10], [11], [12] provides us with an opportunity to (partially) fill the gap in the literature and to study the effects of PR in computer–assisted, constructivist learning by means of an experimental setting. The findings in our previous research [13], [14] suggested that *the cyclical and iterative process of communicating relevant, well–argued and constructive feedback messages by students about the workshop papers of their peers* (this is how we defined PR) were strongly related to the exam scores which measured understanding of statistical concepts, rather than rote memorization. The methodological approach that was employed in our previous studies, allowed us to classify this relationship as “associative” or “predictive” — true causality, however, could not be inferred. It is for this reason, and since we found no hard evidence of causality in our literature search, that it is important to investigate the causal relationship between reviewing peers and deep learning, through an experiment in which the treatment (i.e. the peer reviewing process) is fully randomized and where the treatment effects are not confounded by other factors.

### Technology

#### Reproducible Computing Technology

The concept of peer review-based learning in university-level statistics education is largely uncharted. This may be strange because the need to be able to critically review statistical papers has never been disputed [15]. In passing, the problem of irreproducible research and the problem of providing universally accessible solutions has received a great deal of attention within the statistical community [11]. If statisticians find it difficult (if not impossible) to reproduce the empirical findings reported in scientific papers, then it is unfair to expect students to be able to reproduce, and make sense of, empirical results that are presented in course materials and research papers [15]. It is for this reason that we have been engaged in the development of a novel Reproducible Computing (RC) technology that allows anyone to produce an empirical paper (the so-called “Compendium”) that can be reproduced without the need to install software or the need to understand the underlying statistical technicalities [11]. A more detailed discussion would lead us too far from our central theme; for present purposes it is sufficient to observe that RC can be used to support PR and collaborative work in an educational setting.

#### Peer Review Technology

The implementation of PR in educational practice through online technology has been advocated and studied by several educational researchers [1]. Likewise, in our previous research, we presented pedagogical and technical benefits of the PR technology that we developed, tested, and implemented [12], [16]. The most important and unique feature of the PR technology that we developed, is the fact that it can be seamlessly integrated into other software such that the content (i.e. the document to be reviewed) is unambiguously connected to the feedback that is written by the reviewer. In other words, the feedback becomes meta data of the document that is under review which can be shown to have important consequences in terms of learning efficiency [14].

#### Exchange Technology

We investigated the causal effects of Reproducible Computing (RC) technology [11] and PR learning activities on students' abilities to learn new concepts and apply them within a game-based decision-making environment. The game is based on the Xycoon Stock Exchange (XSE), which is a virtual e-learning environment where students can engage in real trading activities and learn about the economic principles of the stock market and its underlying statistical properties. Unlike other trading games, the XSE engine is based on technology that was originally developed for creating fully functional, web-based stock exchanges and have been used by Euronext [17] and the European Commission [18] for educational and training purposes. All price fluctuations are the result of bid and ask orders that are processed in real-time. The orders are created by the participating students, the educator, and an optional computer trader that is enhanced by Artificial Intelligence, and which relies on heuristic rules from past research of actual trading activities and their relationship with external factors like news messages and various types of economic indicators [10].

We investigate the effects of PR on perceived utility, learning outcomes (about true understanding of the underlying statistical concepts), attitudes towards trading, and the effect on actual trading activities. The rationale behind this is that student's understanding of statistical concepts is insufficient to describe the potential effects of competing learning approaches — changes in actual behavior and attitudes (such as risk aversion) may be equally (if not more) important.

With the exception of perceived utility, all effects are measured by means of objective and accurate observations. This is possible through the use of innovative RC technology and the XSE which have been seamlessly integrated into the learning environment used in this experimental research.

In line with our earlier research which has been focused on reproducibility of statistical computing [11], we made all computations available through hyperlinks that allow the reader to reproduce, re-use, and review our analyses without the need to download, install, or execute any code on the client machine.

## Materials and Methods

### Ethical Considerations

The experiment was conducted with several ethical considerations in mind, which are briefly listed here:

- There was informed consent from the students. All students in this study had the opportunity to indicate whether they wanted to participate in the experiment or not. This was achieved through a selection menu from within the VLE (the choices were stored electronically and could not be forged because the students were required to logon to the VLE). During the lectures, students received detailed information about the experiment. If they chose not to participate, they were required to work on an off-line assignment about an article which covers roughly the same topics as the ones that were introduced in the experiment [19]. The off-line assignment did not involve any randomization, nor any experimental treatment.
- All data were anonymized by replacing student names with unique, non-informative numbers.
- The collected data did not contain any sensitive information.
- The results of the experimental measurements were not used to grade students. Rather, students were graded on their active participation in either the experiment or the alternative (off-line) assignment.
- The experimental treatments under investigation were in no way related to the core statistics curriculum and did not influence student performance at the final examination. In other words, the treatments in the stock market game did not discriminate any students to perform well in the statistics course.
- In most situations, an official approval by an Institutional Review Board (or Ethical Committee) is not required for educational research, as is exemplified by the exemption of “*(i) research on regular and special education instructional strategies, or (ii) research on the effectiveness of or the comparison among instructional techniques, curricula, or classroom management methods”* which is specified by the Federal Policy for the Protection of Human Subjects of the National Science Foundation in the U.S.A. (http://www.nsf.gov/bfa/dias/policy/docs/45cfr690.pdf). Moreover, the applicable law on human experiments (*wet inzake experimenten op de menselijke persoon*, 7 May, 2004, http://ppw.kuleuven.be/onderzoek/ethischecommissie/wet) is explicitly limited to *experiments which develop our understanding of biology and medicine* — in other words, the legislation does not pertain to educational research as is presented in this paper. Notwithstanding the fact that our research is exempt from the traditional ethical review, we would like to point out that our research was funded by an academic agency which involves a series of screening and monitoring procedures, and which is only granted under the condition that there is institutional support and permission to study the pedagogical effects of the technological innovations that are implemented in our experiment (see also Ethics section of [14]).
- The stock market game is part of our extra-curricular offerings. This means that permission to organize the game was granted too.

### Structure of the Course

The experiment was embedded in a compulsory undergraduate statistics course for business students in Belgium. The emphasis of the course was on constructivist learning, based on more than 70 different statistical techniques which cover the following topics: explorative data analysis, hypothesis testing, multiple linear regression, univariate time series analysis, and non-parametric statistics. We used a statistics handbook which was translated from English to Dutch and covers most of the topics of the course [20].

For each technique, students had one or more web-based software modules available within the R Framework which was developed at the University of Leuven [15] and uses the R language [21] on a series of networked servers to compute the statistical analyses. At no time are students required to download, install, or execute any code on their client machines. As a consequence, the system effectively removes the pain of many computational technicalities that might lead to confusion and frustration.

The software is freely available online at http://www.wessa.net/www.wessa.net [22] and features a so-called blogging system that allows students to “blog” (i.e. archive) statistical computations that have been produced in an online repository [11]. Each blogged computation is represented by a unique URL that can be simply inserted into any document. This allows any reader with a live internet connection to consult all the results and associated meta data of the statistical analysis. In addition, the reader is able to reproduce the computation in real-time through the use of the R Framework. It is also possible to change the parameters, data, and software which allows students to challenge (and review) results that are presented in papers from their peers or in course materials provided by the educator.

**Figure 1:**
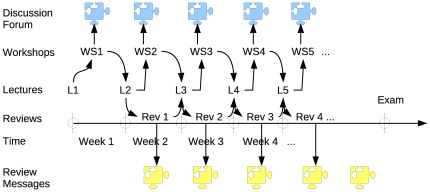
Schedule of learning activities.

In order to implement this course within a setting of constructivism for a large student population, we introduced a strict assignment–review mechanism. This is illustrated in Figure 1 which shows a series of weekly events (lectures, assignments, reviews) during the thirteen–week semester (the horizontal axis represents time). Each week roughly corresponds to one (or two) chapters in the handbook of [20].

The semester ended with a final examination consisting of a series of objective multiple choice questions which referred to a large document containing raw computational output (charts and tables about several data series). The examination was intended to test understanding of statistical concepts rather than rote memorization. More precisely, the exam was designed to test if students were able to:

- identify the computational output that was relevant to the question
- interpret the output in terms of the question
- critically investigate if the underlying assumptions of analyses were satisfied

The main sections of the statistics course were built around a series of research-based workshops (labeled WS1, WS2, …) that required students to reflect and communicate about a variety of statistical problems, at various levels of difficulty. The problems were carefully designed and tested over a long period. Each workshop contained questions about common datasets and questions about individual data series provided to students — this dual structure of the workshops promoted both collaboration between students and individual work. The top (blue) puzzle pieces in Figure 1 represent threaded communication (between students) about each workshop.

Each week there were two (compulsory) lectures which are labeled L1, L2, etc. With the exception of the first and last week, each lecture consisted of the following two parts:

- one or several illustrated solutions of the previous week's workshop assignment based on good and bad examples of archived computations that have been generated by students and the educator
- an introduction to next week's assignment including a reading list and an illustration

During each week, students were required to work on their workshop assignment and — at the same time — write peer reviews (labeled Rev1, Rev2, …) about (an average of) six assignments that were submitted by peers. Each review was based on a rubric of a minimum of three criteria and required students to submit a workshop score and an extended feedback message for each criterion. In Figure 1 these messages are represented by the bottom (yellow) jigsaw pieces.

The PR process was supported by newly developed, innovative software that is based on a so-called content-based design of the Virtual Learning Environment (VLE) which can be shown to be more efficient than traditional PR implementations [14]. The grades that were generated by the peer review process did not count towards the final score of students. Instead, the educator graded the quality of the verbal feedback messages that were submitted to other students. More detailed information about how peer reviews can be assessed, based on our innovative PR technology, is available in the study of [12].

As one might have noted, this feedback-oriented process is similar to the peer review procedure of an article that is submitted to a scientific journal. The process of peer review is an important aspect of scientific endeavor, and may help us in achieving learning goals with respect to attitudes (through peer review experiences) and skills (through construction of knowledge). The key idea behind this constructivist application is that students are empowered to interact with reproducible computations from peers and the educator. Students are required to play the role of active scientists who investigate problems, present solutions, and review the work of peers. Access to web-based *Reproducible Computing* technology is critical in allowing students to engage in such peer review activities.

### Structure of the Embedded Experiment

**Figure 2:**
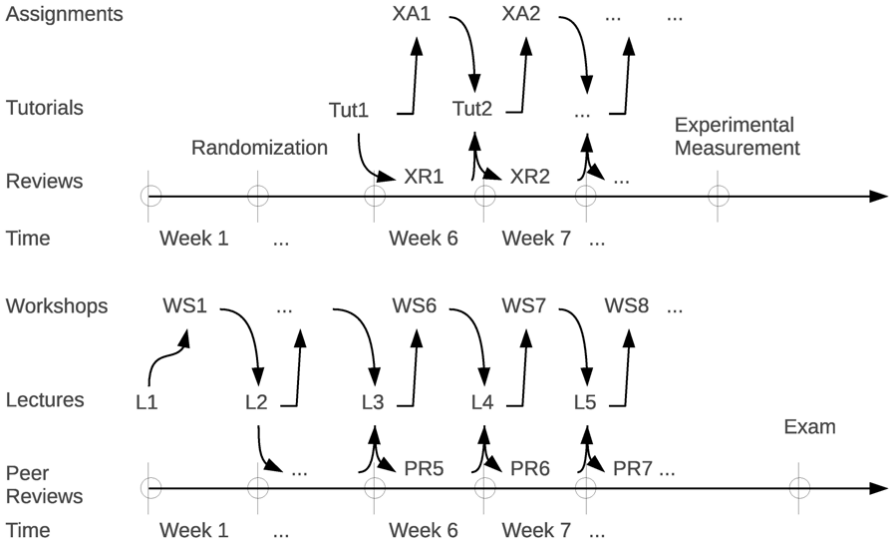
Schedule of experimental learning activities.

The actual experiment was conducted in parallel to the regular course activities as is illustrated in Figure 2. It is important to note that the experiment began several weeks after the start of the regular course in order to make sure that students:

- had sufficient background knowledge of statistical concepts
- had already experienced several rounds of peer review
- were able to use the statistical software and blogging features.

Rather than using regular statistical topics as the subject of experimental study, we opted to use the annual Stock Market Game (SMG), based on the XSE software, as a vehicle to measure learning outcomes. The SMG has a long tradition at several Business Schools in Belgium and the underlying XSE software is stable and thoroughly tested because it was originally developed for EURONEXT and the European Commission, for the purpose of training and research. The participants in the experiment were required to learn about a series of new statistical techniques that can be used to analyze stock market time series and make informed decisions about the investment strategy that is employed.

For instance, one of the assignments that we introduced (XA1, XA2, etc. in Figure 2) treated the difference between an ordinary Random Walk (which corresponds to an “efficient market”) and a Quasi Random Walk (which is typical for a “non-efficient market”). The point of this particular workshop was to introduce the concept that an investment strategy based on a statistical model only makes sense if the stock price time series do not behave as Random Walk processes (see [19] for more information). This clearly illustrates that we required students to learn about statistical concepts (such as the Quasi Random Walk theory) that lie outside of the regular curriculum (in order to avoid discrimination) which, on the other hand, can be accurately and objectively measured based on the XSE software.

The XSE software allows students to interact with the R Framework in real-time. This implies that participants are able to send the stock market time series to any web-based R module for analysis [10]. It is important to understand that the XSE software is not just a simulation of a stock market. On the contrary, it is a real stock market for trading shares among all participants based on ficticious companies. In other words, all stock prices are a result of actual trades (between participants) with rules that are identical to those of the EURONEXT exchange. In addition, and with the purpose of making the market conditions realistic, it is possible to employ a computer trader which is enhanced with Artificial Intelligence. The computer trader simply acts as a so-called “market maker” who constantly maintains orders to buy or sell shares at certain limit prices. This implies that any participant is able to sell or buy shares even if there is no counter order from another player available.

In order to drive trading on the stock market, the game administrator (or educator) is able to influence the news messages that are sent to the traders. If the administrator sends good news about a company into the trading room then there is a good chance that some participants launch orders to buy the shares of that company. In any case, the computer trader, if activated, will respond to the news messages and change its limit prices according to a large number of heuristic rules which are based on our analysis of actual (typical) market reactions that can be associated with similar news messages. The consequence of this mechanism is that the stock prices will fluctuate according to what “normally” happens on the real stock market.

In principle, the administrator is able to steer the market through the manipulation of corporate or general news messages. However, the wealth of the computer trader is limited and can be changed by the administrator. This implies that the influence of orders made by human participants may become much stronger than the impact of the AI-enabled computer trader. In other words, if participants behave irrationally then the stock market prices will show statistical properties that deviate from what could normally be expected [10]. The bottom line is that the administrator has partial control (over the evolution of the stock market prices) to a degree that is determined by the wealth of the computer trader.

The SMG was used to obtain objective measurements of student's ability to apply newly acquired statistical knowledge to solve new and challenging problems. Before the actual measurement was made, participants only knew that they would be required to design a profitable financial investment and implement it through trading activities on the stock market during a period of a few hours. We made the window of measurement relatively short because that ensures that the participants had to work under stress and did not have much time to communicate or collaborate with each other.

During the first weeks of the semester, we introduced the basic concepts of the experiment and also explained the rules of engagement as explained in the Ethical Considerations subsection. In the statistics course there were 314 students who completed the final examination. From this group we had no information about or manually excluded the observations from students who:

- did not want to participate (and chose to do the alternative, off-line assignment)
- were not able to complete the entire experiment (due to illness, etc.)
- dropped out or wished to discontinue the experiment
- did not complete the experimental trading activities within the specfied deadline
- had prior knowledge about the statistics course or the stock market game (e.g, students who had to re-take the course, or played the SMG before)

As a result, we had valid data from a total of 154 students for statistical analysis.

### Market-Neutral Arbitrage Strategy

We announced the date and exact time during which the experimental investment strategy would have to be designed and implemented. The actual description of the challenge however, was unknown to the students and only revealed at the start of the measurement period. Moreover, students did not know beforehand what the market circumstances would be like during the measurement period. In the tutorials (Tut1, Tut2, etc. in Fig. 2) and associated assignments (XA1, XA2, etc. in Fig. 2) the students learned to deal with very specific market situations (for instance, how to analyze the so-called Quasi Random-Walk model as explained in [19]). Before the measurement period began, the administrator changed the market conditions by manipulation of the news messages that were sent into the trading room. This ensured that at the start of the measurement period about half of the traded stocks were rising while the other half was declining. This situtation was new to the students because all the previous tutorials and assignments assumed that most stocks behaved in a similar manner. As a consequence, many of the statistical techniques that were explained in the tutorials were simply invalid (because the underlying assumptions were not satisfied). This is, arguably, one of the most challenging problems for students when learning statistical concepts and therefore a perfect scenario to determine who would be able to pick the statistical techniques for which the assumptions would hold (something which cannot be achieved through rote learning). The most important aspects of the investment strategy to be designed in the game were as follows:

- At the beginning of the measurement period one should analyze the stock market time series and create three piles which are conveniently called: Long, Short, and Neutral.
- We put all the stocks for which we predict an increase onto the Long pile. The stocks which are predicted to decline belong to the Short pile. All remaining stocks are in the Neutral pile.
- When we placed all stocks in the appropriate piles, we buy the shares in the Long pile, and sell the ones in Short pile. Note: on the stock market it is possible to sell shares that one does not already own. In essence one “borrows” the shares from a third party (the broker) and sells them, hoping that prices will fall. At some time in future, the short seller must buy back the borrowed shares (even if the share price has increased). For obvious reasons, short selling is subject to several limitations. Obviously, the stocks from the Neutral pile are not held in the portfolio.
- We hold the Long and Short position until the end of the measurement period. After that we evaluate the profits (or losses) for the investment portfolio.

The above investment strategy is referred to as a “market-neutral arbitrage strategy” (MNAS) which is often used by hedge funds and may be supported by statistical analysis. In theory the MNAS works for “bullish” (rising) and “bearish” (declining) markets as long as one is able to correctly pick the stocks that go into the Long, Short, and Neutral piles. Within the context of our experiment, students had complete freedom to choose how they would make their investment decisions. Since they didn't know that our main interest was in the application of statistical techniques, as it was presented as a trading game, there was no obligation to use any statistical analysis which is illustrated by the fact that some students made their decisions based on economic intuition rather than empirical evidence.

### Statistical Hypotheses

#### Utility Hypothesis

Based on the findings in usability and technology acceptance research, we may expect that our technology-driven approach to constructivist education is affected by several aspects that pertain to students' attitudes and emotional experiences. The study of [23] explicitly examines the causes and effects of perceived usefulness within the context of statistical software adoption. According to their conceptual model, there are several psychological constructs affecting the degree of perceived usefulness, namely:

- statistical anxiety (which is a multi-dimensional concept)
- statistical software self-efficacy
- computer attitude
- perceived ease of use

The perceived usefulness construct, in turn, affects behavioral intentions to use the software in the future. Other studies, such as [24], have approached students' attitudes towards statistics from a “utilitarian” point of view with an emphasis on “utility in studies” and “utility in professional career” as cofactors which explain students' interest and anxiety. The importance of this concept of utility is not only apparent from the academic literature but can also be illustrated with perceived student satisfaction data, which we collected in the past few years through the so-called COLLES survey [25]. The survey contains six subscales which are measured on a 5–point Likert scale: professional relevance, reflective thinking, interactivity, cognitive demand (tutor support), affective support (from peers), interpretation and meaning of messages (from peers and the tutor).

One of the subscales of particular importance for this study is “*Professional Relevance - the extent to which engagement in the on-line classroom environment is relevant to students' professional worldviews and related practices”* [25]. The reason for this is that in the past we have observed relatively low average scores for the the professional relevance subscale in our statistics courses. Based on focus group discussions we have found that most students do not find statistics particularly interesting. This is clearly illustrated by the fact that students perceive statistics as not very relevant for their main field of interest (in this case “business and economics”) which leads to relatively low scores in the practical relevance subscale of the COLLES survey. In addition to that, we computed the difference between the actually perceived and the preferred levels of each item in the survey. Since the preferred levels 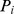 are (on average) higher than actually perceived levels 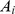, one may interpret the sum of all differences of the four items in the practical relevance subscale 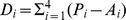 as the degree of dissatisfaction of students with respect to the subject of the course (i.e. statistics).

**Figure 3:**
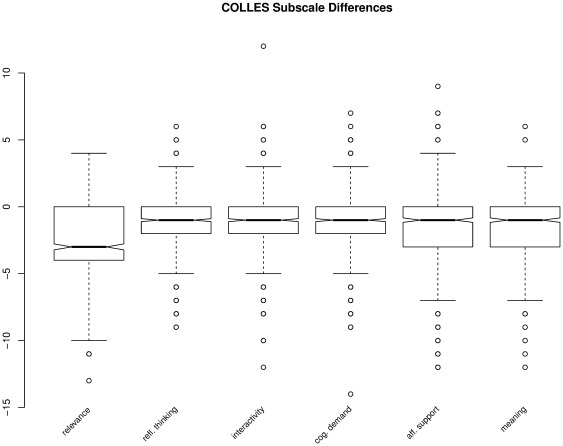
Differences in COLLES subscales (4 consecutive years, N = 804) (www.wessa.net/rwasp_PRcolles.wasp).

Figure 3 demonstrates that in the last four years students were significantly more dissatisfied in terms of the practical relevance subscale than with any other subscale of the COLLES survey. If the subject (of Statistics) is not perceived to be relevant then this may have negative consequences on student education and lead to rote learning. It is also important to note that the professional relevance questions pertain to the perceived utility of Statistics, in a broad sense (not for a small set of particular problems). Most students in this study do not have much experience (if any) with applying statistical techniques to solve practical problems — the relatively high dissatisfaction score should therefore not come as a big surprise.

In an attempt to make Statistics more attractive to students, we have tried to implement a more practical approach than what is done in a traditional or typical statistics course. The constructivist approach to statistics education may seem promising in this regard because it encourages students to experiment, communicate, and experience statistical problems in a more natural or practical environment. It is therefore interesting to investigate whether it is possible to gain students' interest in the subject (of Statistics) through constructivist learning activities such as Peer Review — after all, Statistical Analysis may well be seen as an acquired taste.

Due to its academic and practical importance we have decided to formulate the following null and alternative hypothesis about utility:

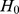
: Peer Review does not cause students to find statistics more useful.

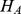
: Peer Review causes students to find statistics more useful.

#### Behavior Hypothesis

The Utility Hypothesis implies that utility or usefulness affects the intention to use statistical software at some undefined time in the future, for the purpose of solving some undefined problem. In other words, this hypothesis implicitly assumes an effect on the long term and for general purposes.

In contrast to this, the study of [26] established that perceived usefulness, among other variables, does not only have an impact on the “intention” to use the software, but also on “actual” use on the short-run for specific learning purposes. Therefore, this so-called “behavioral” impact is different from the “utilitarian” one in terms of the time horizon and the specificity of the problem for which statistical software is used. In addition, the study showed that it is possible to predict actual use by observing constructivist learning activities, such as the submission of PR messages. It is for this reason, and supported by recent academic research about the effects of PR for reviewers and reviewees [7], [8], that we introduced the so-called Behavior Hypothesis:

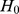
: Peer Review does not cause students to use statistical techniques more often.

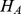
: Peer Review causes students to use statistical techniques more often.

Even if PR does not improve perceived utility of statistics, it still might have an impact on actual use for the purpose of solving particular problems. In addition, it should be noted that the actual (short-run) behavior of students can, unlike percieved utility, be objectively measured because all statistical computations are performed within the R Framework which maintains historical and detailed records of computing activity. If constructivism, by means of PR, is claimed to be beneficial, it should lead to changes in actual behavior on the short-run, even if the problem occurs outside of the regular course (i.e. the SMG).

#### Non-Rote Learning Hypothesis

In line with current tradition in educational research, the pedagogical paradigm of constructivism is believed to support non-rote learning [27]. In our previous research we also found empirical evidence to support the hypothesis that PR has a beneficial effect, most notably for the reviewer [13].

For this reason we treat the Non-Rote Learning Hypothesis as the most most important hypothesis in this study. Even if constructivism (by means of PR) cannot affect behavior or perceived utility, at least we hope to find evidence that it helps students to understand statistical concepts to such a degree that they can solve particular problems with the correct type of analysis (for which the underlying assumptions are satisfied).

We specify the non-rote learning hypothesis as follows:

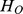
: Peer Review does not cause students to adequately apply the correct type of analysis.

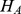
: Peer Review causes students to adequately apply the correct type of analysis.

The literature review of [27] is important in this respect because it provides an excellent overview of the factors that encourage or discourage the effectiveness of deep-learning approaches within the context of student-centered learning environments. In their review they stated that: “*The results of the studies addressing the effects of student-centred learning environments on students approaches to learning were not univocal.”*. The effects of receiving feedback from the instructor or through PR in particular, does not seem to be unambiguous either. Furthermore they found that “*These mixed findings make clear that influencing students approaches towards deep learning by means of implementing student-centred learning environments is a complex process. Numerous other factors that encourage or discourage the adoption of a deep approach may be of influence.”* — this finding illustrates the relevance and our motivation of investigating the pure effect of submitting PR messages based on a randomized experiment.

#### Attitude Hypothesis

Learning outcomes in academic education are not only expressed in terms of skills (as described in the Non-Rote Learning Hypothesis) but also relate to attitudes. In the curricular definition of our academic courses it is often specified what type of attitudes should be changed or improved. In daily practice however, one rarely sees any evidence that a course truly affects student attitudes, let alone that attitudes would be estimated through the use of surveys or based on objective measurements. In our experiment, we had the opportunity to investigate this matter based on objective measurements of trading actions which are closely related to students' attitudes.

Within the XSE software, students could submit orders to buy and sell shares according to the rules of the EURONEXT exchange. One of those rules specifies that traders have the option to submit Market Orders (MO) or Limit Orders (LO). A MO is simply a request to buy or sell shares in a certain quantity. The price at which the trade should take place is not specified by the submitter of the MO. Therefore, the exchange will search for the “best” counter party that is currently available. The price at which the trade is executed is simply the “best bid” (highest bid price) or “best ask” (lowest ask price) of all available counter parties. On the contrary, the LO allows the trader to specify a quantity and a limit price. For instance, if the trader wishes to buy shares at a limit price of EUR 10 per share, then the order will only be executed if there is a counter party with a MO or a LO which specifies a selling limit price that is not higher than EUR 10.

In the experimental tutorials it is clearly explained how the order system of the stock exchange works and how this is related to what is commonly called “market liquidity” (i.e. the property that ensures that shares can be sold or bought quickly and without large price changes). As explained before, the XSE is not a simple simulation of stock prices — it *is* a real stock market where prices are determined by the interplay between bid and ask orders. The role of the computer trader was kept limited on purpose — this was achieved by making sure that the computer trader had the equivalent wealth of roughly five human players. Remember that we required students to implement the MNAS within a relatively short time frame of a few hours (on a Friday afternoon). This had several important consequences for the stock market and its liquidity:

- many human, and relatively inexperienced, participants would enter the market at roughly the same time
- if all human traders make the same decisions there will be no counter party available (the counter offer from the computer trader would soon be completely executed which leads to a situation where the best counter offer is made by another human participant and which may well have an extreme limit price)
- some (smart) participants submitted buy and sell LOs at extreme prices, knowing that in times of stress, many traders would simply submit MOs. These participants are literally hoping that chaos occurs because that would cause them to make large profits.

Students did not know before or during the experiment how large the impact of the computer trader would be. They also did not know that their choice of order (MO or LO) was of particular interest in our experiment. In other words, there was no indication or information about the importance of MOs versus LOs that could have affected the outcome of the experiment. In addition, it is important to understand that students did not only learn about statistical techniques, but also about the statistical properties of the stock market and how this affects traders. Only those students who would have fully understood the mechanism of the stock market and its statistical properties would have had the opportunity to learn or acquire the attitude that trading during the MNAS implementation period would be potentially dangerous.

The attitude hypothesis is formulated as:

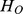
: Peer Review does not cause students to be cautious and use Limit Orders more often.

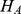
: Peer Review causes students to be cautious and use Limit Orders more often.

We defined the statement “to use Limit Orders more often” according to the ratio 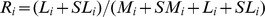 for 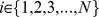 where

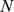
 is the number of students

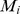
 is the number of (ordinary) Market Orders of student 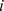

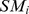
 is the number of Stop Market Orders of student 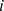

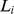
 is the number of (ordinary) Limit Orders of student 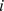

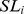
 is the number of Stop Limit Orders of student 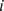

Whenever 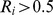 we assigned the label “Yes” to the variable “UseLimit” in the database. The label “No” was used for students where 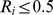.

#### Outcome Hypothesis

During the preparations of the experiment we did not know whether our intended illiquidity would work or not. In other words, we were uncertain whether the fluctuations on the market would be most strongly affected by the students or the computer trader. Based on the AI rules in the computer trader software, we knew that under normal circumstances (i.e. the situation where students would not have a dominant impact on prices) certain stocks would rise and others would fall. As a consequence, the outcome (in terms of profit) of the MNAS investment strategy was known under the condition that students' impact on prices would not be dominant. It is therefore interesting to investigate whether the PR treatment would cause students to achieve higher profits or not.

The outcome hypothesis is as follows:

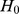
: PR does not cause students to yield better trading results.

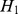
: PR causes students to yield better trading results.

On the other hand, if students would dominate the price fluctuations on the market, the outcome of any rational investment strategy would be highly uncertain and contaminated by irrational behavior from inexperienced participants, as is predicted by [28]. Consequently, the outcome hypothesis does not make sense in this scenario.

### Treatments and Timeline

#### Peer Review and Cohorts

The treatment under investigation is PR or more precisely, the submission of PR feedback messages to other students. As is explained in the empirical analysis of [13] the main benefits of PR are expected to be observed from the perspective of the reviewers, not the reviewees. This is in agreement with recent literature as described in [7] and [8].

It is for this reason that we embedded the same feedback mechanism in the experiment as was used in the regular course. There was only one crucial difference: the students in the randomly selected control group did not participate in PR but received ordinary feedback from the educator. Additionally, the control group students were required to correct mistakes from the previous workshop, which was to be submitted together with the next one. In other words, the control group followed an ordinary cycle of feedback as is encountered in many courses. Other than that there was no difference between the control and treatment groups. The assignments were identical and all students were assigned completely at random, which implies that measured differences (the so–called effects) can be interpreted in terms of causality.

Based on personal experiences and (unpublished) preliminary research, we believe that PR is only beneficial when it is applied frequently and for a longer period of time. This hypothesis is in line with our conclusions from focus group discussions in which students reported that PR is a “new learning method” which requires time to get used to. Our estimate was that a consecutive series of (at least) three rounds of PR would be necessary to obtain a beneficial effect. For this reason, we decided to conduct the experiment for two different cohorts: one with 2 full rounds of PR about large assignments, and one with 4 full rounds of PR about medium-sized assignments. It is our expectation that the treatment effect of PR would work at least as good, if not better, in the 4–round group as compared to the 2–round group. As a consequence, each of the five hypotheses is examined for each of the two cohorts, yielding a total of ten statistical hypotheses.

#### Timeline

The timeline of the experiment is outlined briefly because it has important reprecussions to understand the results of the experiment. There are three phases in the experiment which are conveniently labeled A, B, and C.

**Figure 4:**
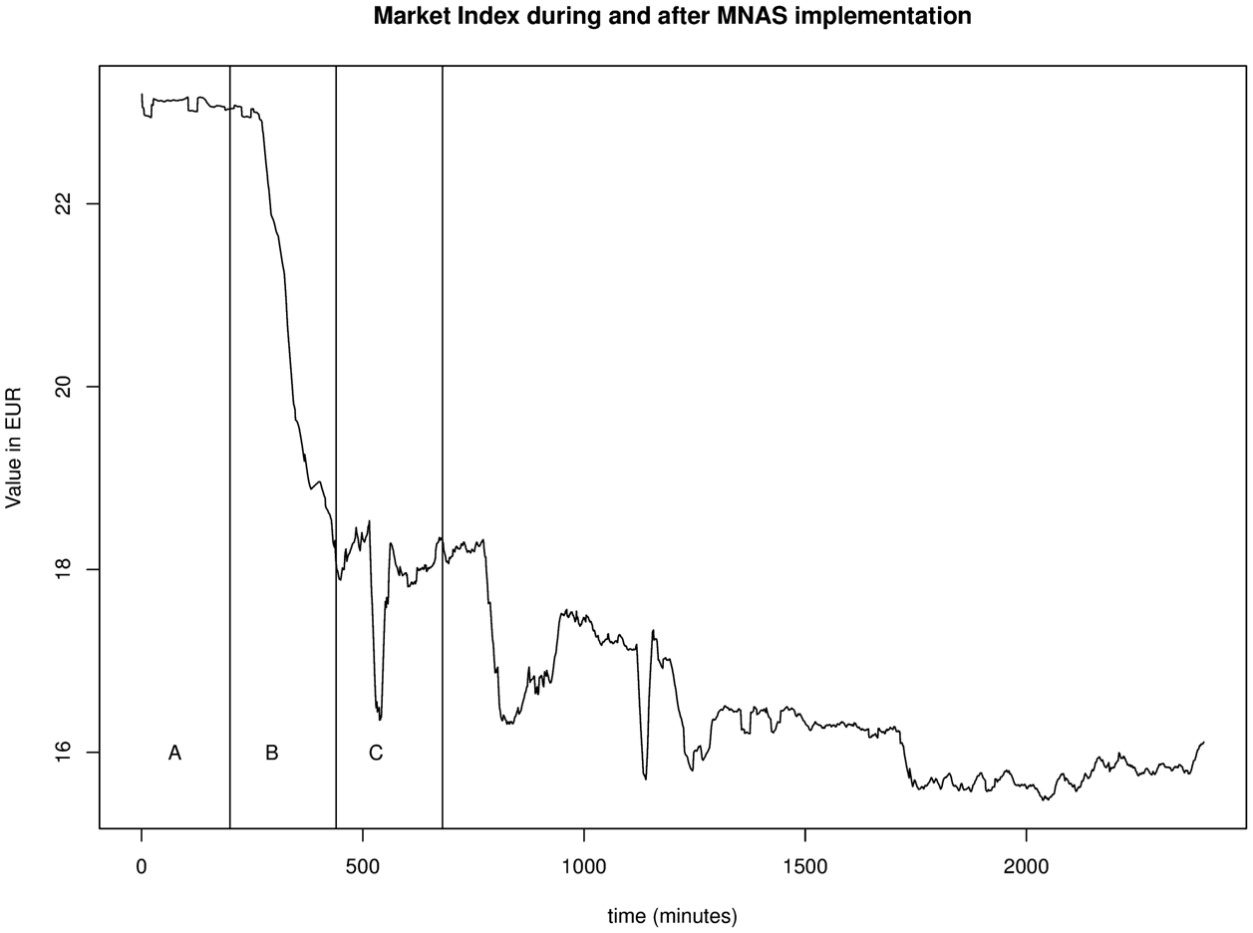
Market Index as a result of MNAS implementation (www.wessa.net/rwasp_PRMNAS.wasp).

Phase A is the preparation period which was needed to ensure that the stock market's statistical properties are perfect to perform a MNAS. More precisely, the news messages were manipulated by the game administrator such that half of the companies' stock prices were (slowly) rising and the other half was (slowly) decreasing. The overall stock market index was neither bullish nor bearish and displayed a flat line as can be seen in panel A of Figure 4. The preparation period was long enough for students to be able to empirically detect the underlying statistical properties (the actual time is longer than what is shown in the Figure).

At the end of phase A, students received detailed information about the task they had to perform. There was not enough time to start collaborating because students were required to specifiy their investment decisions at the start of phase B. Again, students were not required to use statistical techniques — they had complete freedom to make their decisions. However, any student who wished to use statistics had no other data available than the historical prices of phase A and the associated news messages. In other words, students had every (statistical) reason to believe that circumstances during phase B would remain the same as in phase A (in Economics this is called the “ceteris paribus” condition).

On the other hand, students also knew that a large group of peers would be implementing the MNAS during phase B. They knew, based on economic theory outlined in the tutorials, that this could have consequences for the statistical properties of the stock market. It was therefore important to stay online during phase B and to use LOs instead of MOs. The instructions for students clearly indicated that they were required to:

- determine the stocks that went into the Long, Short, and Neutral piles
- to submit the buy and sell orders *at the beginning of phase B*
- not change the portfolio during phase B (as a consequence of new information that would become available)

This implies that the measurements of the experimental outcomes for the Behavior, Non-Rote Learning, and Attitude Hypotheses are made, based on the actions during the start of phase B. The actual change of the market index during phases B or C is entirely irrelevant. Only the Outcome Hypothesis could be affected by the actual events during phases B or C (for instance if the stock market would behave erratically).

Phase C was intented to provide students with an opportunity to trade freely, without any restrictions. Students were allowed to liquidate the MNAS portfolio and change their investment strategy. The students knew that we would be interested in the accumulated profits/losses at the end of phase C. For this reason, many students continued trading activities in an effort to improve their performance, even though this did not count for the grades they received. As explained before, the outcome hypothesis only makes sense if the stock market behaves (more or less) rationally during phases B and C.

### Statistical Analysis

Fisher's Exact Test (FET) is appropriate for the analysis of our experimental study. The underlying assumptions of the FET are the same as for traditional 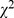 tests, with the exception of the assumption that the expected frequencies should be sufficiently large [29]. The knowledge that some expected cell frequencies may be rather low, makes the FET a far better choice for testing the statistical hypotheses.

Why is it that we expect low frequencies in certain cells of the contingency table? The reason is related to the way the experiment was conducted:

- Roughly half the student population was randomly assigned to the PR treatment group (the other half forms the control group).
- Not all students in the PR and Control groups were actively participating in the experiment. For this reason, we measured the degree of activity of all students through objective, quantitative observations which were collected though the RC technology and the XSE. We discarded the data of all students who did not actively participate from the dataset.
- Some of the experimental measurement frequencies are expected to be low. For instance, the correct application of statistical techniques to investigate and implement the MNAS strategy, is rather difficult to achieve for our student population. We know this because the MNAS strategy used to be thaught in another course in the past, for a student population which is very similar.

One of the implicit assumptions of the FET is that the row and column sums are predetermined by the researchers [29]. While this is a rather mild assumption, it is still interesting to note that we were able to predetermine the row/column sums with a reasonable approximation, based on the statistics of student participation in the regular course which had been already en-route for several weeks. It is because of this predetermination that we decided to reduce the number of treatments and cohorts from that originally planned.

In our first draft for the experimental design, we intended to use four different cohorts each of which would have been subdivided into four randomized treatment groups: the maturationist group (having access to RC and PR but without any guidance from the educator), the worked-example group (with access to RC but not PR), the constructivist group (with access to RC, PR, and educator guidance), and the control group. However, when we examined the statistics from active student participation in the regular course, we were able to estimate that an experimental design with 4 different 2×2 tables would have resulted in row and column sums which were too low to have reasonable confidence, even when the FET analysis is used. After all, the fact that FET analysis works for “small samples” does not imply that one will be able to estimate the treatment effects with sufficient accuracy. Hence, we decided to reduce the design to two different 2×2 tables for each hypothesis X — the structure is outlined in Table 1.

**Table 1:** Structure of Experimental Contingency Tables.

Hypothesis X						
	2 rounds of PR			4 rounds of PR		
	No Effect	Effect	Total	No Effect	Effect	Total
No Treatment	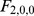	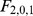	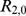	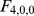	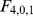	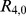
Treatment	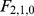	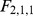	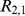	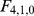	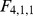	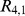
Total	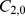	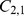	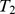	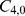	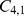	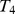

Due to the reduction of the number of treatments and cohorts we were fairly confident (before the start of the experiment) that 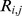 and 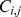 for 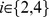 and 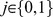 of Table 1 would be high enough. Even though our estimates of 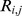 and 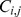 where not exact, it turns out that our approximation did not deviate much from the actual outcomes. It is therefore, reasonable to assume that all FET assumptions are, indeed, satisfied. However, in order to satisfy even the most critical readership, we decided to additionally report the traditional Likelihood Ratio (LR) 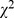 and the Pearson 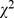 for comparison purposes.

Another reason why the FET is an appropriate choice of test, is the fact that it is possible to use the Odds Ratio (OR) which can be easily interpreted and tested statistically (with confidence intervals and p-values) within the R language which is used in the RC technology. The OR is simply the odds of success in the treatment group relative to the odds of success in the control group. Hence, it provides us with an effect size that is easily understood: the OR simply states how much more likely it is to obtain the desired outcome when the treatment is applied as compared to the situation when the treatment is not applied. It is therefore obvious that the treatment is beneficial when the OR is (much) larger than one. The statistical hypothesis test is performed against the Null Hypothesis that 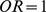. The Alternative Hypothesis is that 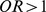 at the chosen type I error — we use a one–sided test because it would be unreasonable to assume that PR would have an adverse effect, especially when the empirical evidence from prior studies is considered [13].

### Data

There are three datasets in this study which are available online (www.wessa.net/download/PRexperiment/experiment.csv, www.wessa.net/download/PRexperiment/marketindex.csv, and www.wessa.net/download/PRexperiment/colles.csv) in “comma separated values” format which can be imported into any modern spreadsheet. The first file contains the data from the randomized experiment with the participants in rows and the variables in columns. The dataset has been cleaned (drop-outs and inactive students were removed) and is readily available for analysis. The second file represents the time series of the stock market during phases A, B, and C. The third file contains the data from the COLLES survey which was collected over a time span of four consecutive years.

## Results

### Stock Market Crash

As explained before, *ceteris paribus*, one would expect that the market index of the stock market would remain flat (as was the case in phase A). The most remarkable result during phase B of the experiment however, was the fact that stock prices crashed (see Figure 4). There was no (statistical) reason whatsoever for this occurence and it would probably not have happened in a market with professional traders. The crash in our experiment was caused by students who did not fully understand the underlying laws of economics and statistics. Through a combination of (excessive) use of MOs and unfounded decision–making, stock prices soon started to decline (even for the companies with positive news messages). The computer trader was able to play the role of market–maker and acted as counter party for orders during the first few minutes of phase B. After that initial period, human players' influence became dominant which resulted in a disasterous crash of all stock prices. Since the implementation of the MNAS was a “difficult” task, many students submitted orders without a real (deep) understanding of the underlying concepts. It is primarily this group of rote–learners' fault that the market crashed — based on the written feedback from students we know that many of them simply learned to use the trading system (i.e. how to submit an order) without truly understanding why an order should be placed or how limit prices could be determined.

During phase C, students were allowed to trade freely. In an attempt to make up for the massive losses that were incurred during phase B, many students continued trading activities, which was often accompanied with risk taking. As a result, phase C was very volatile even though there were no reasons for high volatility in the news messages that were still sent into the trading room. Something which is even more remarkable is the observation that after the end of phase C (this is also the end of the official experiment), trading activitites were still much higher than during the pre–experiment period. Many students continued trading even though this was not expected of them, nor did they get any credit for participating in trading after the experiment. The post–experiment period clearly shows a continuation of high volatility which slowly converges to “normal” levels.

### Hypotheses Tests

Table 2 displays all relevant statistical results which allows us to examine the hypotheses that have been formulated. Each hypothesis is briefly discussed in turn.

**Table 2:** Fisher's Exact Test for Count Data (www.wessa.net/rwasp_PRexperiment.wasp).

Utility Hypothesis: does PR cause students to find statistics more useful?		
	2 rounds of PR	4 rounds of PR
Odds Ratio	0.6262178	1.643887
OR 95% CI	[0.08793619, Inf[	[0.640524, Inf[
OR p-value	0.8272	0.2238
LR 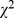	0.34442	1.0017
Pearson 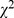	0.32536	1.0210

*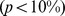;

**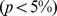;

***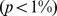.

#### Utility Hypothesis

The Utility Null Hypothesis is not rejected in both cohorts of the experiment. This implies that there is no evidence that PR causes students to perceive statistics as more generally and practically relevant. It does however, not imply that there is no causal relationship. The hypothesis testing framework only works in a confirmatory way and cannot be used to dismiss an alternative hypothesis entirely.

In addition, it is also interesting to note that the OR increases (while the p-value decreases) when we change the number of PR cylces from 2 to 4. While this does not allow us to conclude anything definitive, it is still consistent with the hypothesis that PR could affect perceived utility on the long-run. Maybe we need even more than 4 rounds of PR before a significant effect can be measured — this would not be surprising because students often associate solutions (in this case statistical analysis) with very specific problems, not general ones. Only after many examples, and after a long time, one may realize that statistical solutions are generally and practically useful.

#### Behavior Hypothesis

Both experimental cohorts show a significant impact of PR on the actual use of statistical techniques. The effect in the 2 round cohort seems to be larger than in the 4 round cohort which is probably due to the fact that overall levels of usage (of statistical techniques) in the 4 round cohort was substantially higher. In other words, a relatively higher proportion of non-treatment students in the 4 round cohort used statistics than the non-treatment students in the 2 round cohort. Hence the increase which is caused by PR in the 4 round cohort is smaller and the best reason to explain this is the fact that students in the non-treatment group have more opportunity to experiment with statistical techniques when learning takes place in smaller and more frequent assignments.

#### Non-Rote Learning Hypothesis

From the results it can be concluded that PR causes deep (non-rote) learning within the cohort with 4 rounds of review. The OR is large and implies that students with PR are (almost) seven times more likely to use the appropriate statistical analysis than students who experienced traditional feedback. There is no benefit from PR in the 2 round cohort which does not come as a surprise for reasons that were explained before. Both results seem to suggest that three consecutive rounds of PR is a threshold for the beneficial effect to occur. It is also possible that the effect grows with the number of PR cycles — this however, is a hypothesis that would require more research.

#### Attitude Hypothesis

During the design phase and preparation of the experiment, we did not believe that the null of the attitude hypothesis would be rejected. We would have been happy to find only a significant impact of PR on non-rote learning — as it turns out however, PR *does* cause students to be more cautious which implies that PR could lead to long-term effects.

The results clearly demonstrate that in the 4 round cohort, PR causes students to use LOs more often than MOs. This is not the case for the 2 round cohort which supports the hypothesis that PR is only beneficial when it is applied frequently.

#### Outcome Hypothesis

This hypothesis has become obsolete because of the erratical price changes during phases B and C which were caused by the students. We anticipated (or even hoped) that this would happen before the start of the experiment, even though this would invalidate the results for the outcome hypothesis. The reason why it was still interesting to maintain this hypothesis has two important reasons:

- We did not know for sure that the crash would occur. Therefore, it was still scientifically appropriate to formulate the hypothesis.
- There is now compelling evidence that true understanding of the underlying statistics and economics is relevant and may have serious repercussions for the behavior of financial markets. Future generations of students may now see the consequences of rote-learning.

### Binomial Effect Size Displays

The ORs in Table 2 can also be presented in terms of the so-called Binomial Effect Size Display (BESD) as suggested by [30]. Table 3 shows the BESD for the significant Odds Ratios and can be interpreted as the frequencies one would obtain if half of the population would receive the treatment, and half of the population would exhibit the desired effect [29]. Even though care should be taken when interpreting the BESD in the presence of assymetry in the raw contingency tables (as is explained in [31]) Table 3 provides, nevertheless, an intuitive indication of effect size.

**Table 3:** Binomial Effect Size Display (www.wessa.net/rwasp_PRexperiment.wasp).

Behavior effect: PR increases the use of statistical analysis						
	2 rounds of PR			4 rounds of PR		
	No Effect	Effect	Total	No Effect	Effect	Total
No Treatment	68.6	31.4	100	59.8	40.2	100
Treatment	31.4	68.6	100	40.2	59.8	100
Total	100	100	200	100	100	200

Non-rote learning effect: PR causes students to choose the correct analysis						
	2 rounds of PR			4 rounds of PR		
	No Effect	Effect	Total	No Effect	Effect	Total
No Treatment	–	–	100	65.4	34.6	100
Treatment	–	–	100	34.6	65.4	100
Total	100	100	200	100	100	200

Attitude effect: PR causes students to use Limit trades more often						
	2 rounds of PR			4 rounds of PR		
	No Effect	Effect	Total	No Effect	Effect	Total
No Treatment	–	–	100	63.4	36.6	100
Treatment	–	–	100	36.6	63.4	100
Total	100	100	200	100	100	200

## Discussion

Without a doubt, PR is one of the more important learning tools that is offered by the pedagogical paradigm of constructivism. In spite of the many empirical studies that touch on the importance of PR, there is little or no (hard) evidence for the hypothesis that PR leads to non-rote learning. More importantly, there seems to be a tendency to neglect the fact that PR may have completely different implications for reviewees and reviewers. This difference is explicitly taken into account in our attempt to answer this research question by comparing control group students with normal instructor-based feedback versus treatment students who are actively submitting feedback to their peers. In addition to this, there is, based on our fully randomized experiment, compelling evidence that the submission of PR feedback causes deep learning (Non-Rote Learning Hypothesis), changes the actions that are undertaken to solve specific problems under uncertainty (Behavior Hypothesis), and impacts attitudes which may lead to different behavior on the long run (Attitude Hypothesis). These effects are not only statistically significant but also substantial in terms of their underlying OR and Binomial Effect Size.

The Outcome Hypothesis was obsolete due to the stock market crash that was caused by the students in the aftermath of the MNAS implementation — a pure consequence of irrational behavior on the part of a substantial proportion of the student population with little experience and understanding of the underlying concepts from economics and statistics. As a consequence, we were not able to demonstrate improved investment outcomes in the treatment group as compared to the control. On the other hand, the crash was predicted by economics [28] and clearly illustrates the practical relevance of sound and well-founded statistical analysis.

There are good reasons to believe that the unfavourable perception of the practical relevance of statistics is an important source of potential dissatisfaction which may lead to rote learning. Unfortunately, we were not able to confirm that the PR treatment improves students' perception of relevance — which however, does in no way imply that there is no impact. As a matter of fact, it can be observed that the OR in the 4-round group treatment group is higher than in the 2-round group (while the corresponding p-value drops from 0.83 to 0.22). It is still possible that PR *does* cause improved relevance perception — however, this causal relationship may not be measurable with only 4 full rounds of PR. Therefore, we maintain our belief in the long-run impact of submitting PR on students' perceived relevance, something which may well deserve more in-depth study in the future.

Finally, we would like to point out that the experimental design in this study, while classical and straightforward, is characterized by several unique features that strengthen our confidence in the results that are portrayed. Firstly, and with the exception of the Utility Hypothesis, all experimental observations are based on objective measurements that were generated by innovative, educational technology. This not only improves our confidence in the quality of the data, but it also allows us to gain much stronger control over the circumstances in which the experiment is conducted (precise timing, ability to deny certain features to some groups, detection of inactive students, etc.). Secondly, the experiment is embedded in a challenging game which has a history of many years and is known to be enjoyable and captivating. This is illustrated by the fact that intensive trading activities continued to be observed even after the experiment had ended and is likely to have contributed to the success of the experiment. It is our assertion that the game setting contributed to the students' motivation to perform well and to make the right decisions. Last but not least, the measured learning outcomes lie outside of the regular curriculum which implies that the challenge students faced was to solve an entierly new problem which is situated in a realistic environment and for which students had no textbook cooking recipe that could be applied. This ensured that the learning outcomes can be truly interpreted as insights that have been acquired through non-rote learning, rather than plain memorization of facts and theories. In addition, this aspect of the experiment also ensures that there was no discrimination towards the students in the control group because the learning outcomes of the experiment did not count towards the final results of the stats course.
